# Integrins are required for tissue organization and restriction of neurogenesis in regenerating planarians

**DOI:** 10.1242/dev.139774

**Published:** 2017-03-01

**Authors:** Florian Seebeck, Martin März, Anna-Wiebke Meyer, Hanna Reuter, Matthias C. Vogg, Martin Stehling, Karina Mildner, Dagmar Zeuschner, Franziska Rabert, Kerstin Bartscherer

**Affiliations:** 1 Max Planck Research Group Stem Cells & Regeneration, Max Planck Institute for Molecular Biomedicine, Von-Esmarch-Str. 54, Münster 48149, Germany; 2 Medical Faculty, University of Münster, Albert-Schweitzer-Campus 1, 48149 Münster, Germany; 3 Flow Cytometry Unit, Max Planck Institute for Molecular Biomedicine, Röntgenstrasse 20, Münster 48149, Germany; 4 Electron Microscopy Unit, Max Planck Institute for Molecular Biomedicine, Röntgenstrasse 20, Münster 48149, Germany

**Keywords:** Regeneration, Integrins, Stem cells, Planaria, Patterning, Ectospheres

## Abstract

Tissue regeneration depends on proliferative cells and on cues that regulate cell division, differentiation, patterning and the restriction of these processes once regeneration is complete. In planarians, flatworms with high regenerative potential, muscle cells express some of these instructive cues. Here, we show that members of the integrin family of adhesion molecules are required for the integrity of regenerating tissues, including the musculature. Remarkably, in regenerating *β1-integrin* RNAi planarians, we detected increased numbers of mitotic cells and progenitor cell types, as well as a reduced ability of stem cells and lineage-restricted progenitor cells to accumulate at wound sites. These animals also formed ectopic spheroid structures of neural identity in regenerating heads. Interestingly, those polarized assemblies comprised a variety of neural cells and underwent continuous growth. Our study indicates that integrin-mediated cell adhesion is required for the regenerative formation of organized tissues and for restricting neurogenesis during planarian regeneration.

## Abstract (precis)

**Highlighted article:** Integrin signaling acts to recruit and localize progenitor cells following injury, thereby promoting the correct organization of regenerating planarian tissue.

## INTRODUCTION

Tissue regeneration requires tight control of cell proliferation, differentiation, migration and patterning processes, giving rise to new cells and organizing them into tissues. The planarian flatworm *Schmidtea mediterranea* represents a powerful model organism in which to study these processes as it is capable of regenerating any missing body part, even the head, after injury (Owlarn and Bartscherer, 2016). The cellular source for the construction of new tissues is a large heterogeneous pool of adult stem cells called neoblasts, which are the only proliferative cells in planarians (Baguñà et al., 1989; Bardeen and Baetjer, 1904; Dubois, 1949; Lender and Gabriel, 1965; Randolph, 1892; van Wolfswinkel et al., 2014; Wagner et al., 2011; Wolff and Dubois, 1948). After amputation, neoblasts proliferate, accumulate at the wound site and give rise to a regeneration blastema (Wenemoser and Reddien, 2010). Within the blastema, neoblast progeny form new tissues under the influence of patterning signals (Hill and Petersen, 2015; Scimone et al., 2014b; Vogg et al., 2014). It is likely that subepidermal muscle cells are the source of these signals as they express different sets of patterning genes [also called position control genes (PCGs)] depending on their position in the body. Importantly, they are capable of adjusting their gene expression profiles to wound types (Witchley et al., 2013) and levels of *β-catenin* expression (Reuter et al., 2015; Scimone et al., 2016), a major player in the Wnt signaling pathway controlling patterning along the anterior-posterior body axis (Gurley et al., 2008; Iglesias et al., 2008; Petersen and Reddien, 2009). The planarian musculature might therefore constitute a coordinate system for informing neoblasts and their progeny about their relative position within the tissue (Scimone et al., 2016; Witchley et al., 2013).

Here, we show that members of the integrin family of adhesion molecules are required for organized tissue formation, including the musculature, in regenerating planarians. Interestingly, *β1-integrin* RNAi planarians not only regenerated mispatterned tissues but also displayed increased numbers of mitotic cells and progenitor cell types, and they developed ectopic neural structures (‘ectospheres’). Our study demonstrates the importance of integrin adhesion molecules for tissue patterning during regeneration and suggests that neoblast behavior strongly depends on their communication with an intact extracellular environment.

## RESULTS

### Altered neoblast behavior in regenerating planarians after *β1-integrin* depletion

**Fig. 1.**
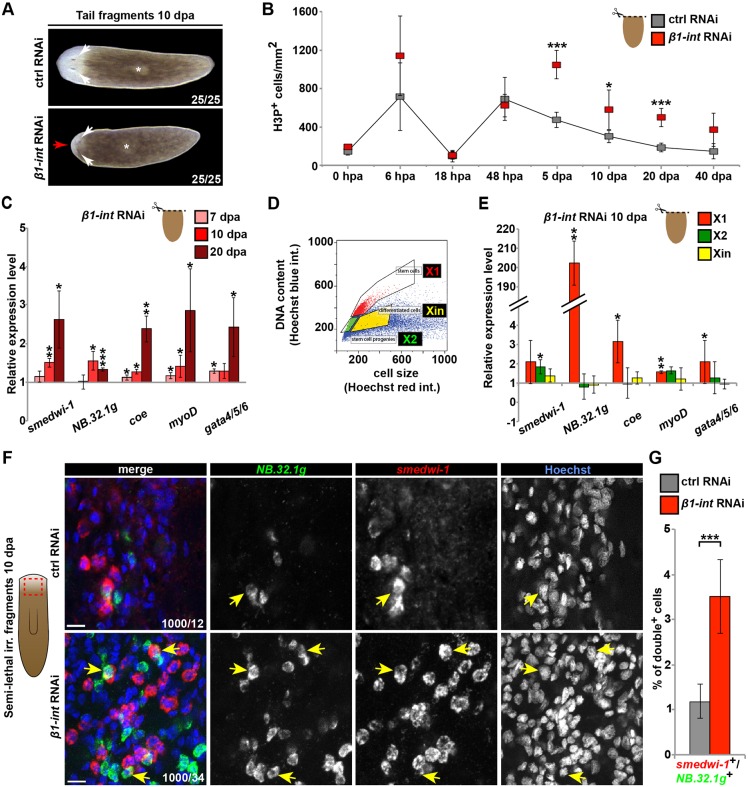
**Impaired regeneration and altered neoblast behavior in regenerating *β1-int* RNAi planarians.** (A) Control (ctrl) and *β1-int* RNAi tail fragments at 10 days post amputation (dpa). Red arrow points to small regeneration blastema, white arrows to regenerated eye spots; asterisks indicate regenerated pharynges. (B) Number of H3P^+^ cells in regenerating ctrl and *β1-int* RNAi tail fragments. Error bars represent s.d. of at least seven fragments. (C) qPCR analysis of indicated marker genes in ctrl and *β1-int* RNAi tail fragments. Expression levels in *β1-int* RNAi fragments were normalized to the corresponding ctrl RNAi samples. Error bars represent s.d. of three biological replicates with ten fragments each per condition. (D) Scheme of cell fractions isolated by FACS according to size and DNA content for gene expression analysis in E: X1 (red; neoblast with 4N DNA), X2 (green; neoblasts/small progeny with 2N DNA) and Xin (yellow; irradiation-insensitive postmitotic cells). (E) qPCR analysis on FACS-sorted planarian cell fractions from tail fragments. Expression levels in *β1-int* RNAi cell fractions were normalized to the corresponding ctrl RNAi samples. Error bars represent s.d. of three biological replicates with sorted cells from 30 fragments for each condition. (F) Double FISH against *smedwi-1* (red) and *NB.32.1g* (green) indicates an increase in *smedwi-1*^+^/*NB.32.1g^+^* cells (yellow arrows) in *β1*-*int* RNAi animals at 10 dpa and 11 days post sublethal irradiation (12.5 Gy). DNA is blue (Hoechst). Red box in scheme indicates imaged area in animals. (G) Evaluation of double *smedwi-1*^+^/*NB.32.1g*^+^ cells in ctrl and *β1*-*int* RNAi fragments. Error bars represent s.d. of counted cells from seven animals per condition. Statistical significance (Student's *t*-test) is indicated (*P≤0.05; **P≤0.01; ***P≤0.001). Scale bars: 10 µm.

Integrin adhesion proteins facilitate interactions between cells and the extracellular matrix (ECM) and hence promote tissue stability, cell migration and a stable cellular environment for stem cells (Boudreau and Jones, 1999; Ellis and Tanentzapf, 2010; Gumbiner, 1996). Based on sequence similarity to vertebrate integrins we identified five integrin genes in *S. mediterranea*. Whereas four of these (*Smed-α-integrin-1-4*; *α-int-1-4*) were similar to α-integrin type proteins in terms of predicted protein domains and sequence similarity (Fig. S1A-D,F), one (*Smed-β1-int*) was classified as a β-integrin family member (Fig. S1E,F). We analyzed a potential requirement for integrin-mediated processes during regeneration using RNA interference (RNAi) (Fig. S2A). Despite their ability to regenerate eyespots and pharynges, fragments of *β1-int* RNAi animals had smaller blastemas at 10 days post amputation (dpa) (Fig. 1A; Fig. S2B). Integrins form heterodimers composed of one α- and one β-subunit to generate functional transmembrane receptors (Campbell and Humphries, 2011). Hence, knockdown of the only planarian β-integrin subunit should eliminate integrin receptor function. Whereas we did not detect obvious RNAi phenotypes for *α-int-1*, *-3* and *-4*, *α*-*int-2* RNAi planarians revealed regeneration defects similar to *β1-int* RNAi animals (Fig. 1A; Fig. S2B,C). This suggests that β1-INT/α-INT-2 heterodimers might be important for regeneration in planarians. We found both *α-int-2* and *β1-int* genes expressed ubiquitously in intact planarians, with *β1-int* expression being particularly strong in the parenchyma, where neoblasts reside, and in the brain region (Fig. S2D,E).

Reduced blastema size might indicate defects in neoblast proliferation, differentiation (Reddien et al., 2005a) or migration to the wound site. Interestingly, *β1-int* expression was strongly reduced 2 days after γ-irradiation, an efficient method of depleting neoblasts (Bardeen and Baetjer, 1904; Reddien et al., 2005b). We also detected *β1-int* transcripts in neoblast-containing cell populations sorted by fluorescence-activated cell sorting (FACS) (Hayashi et al., 2006), suggesting expression of this gene in neoblasts (Fig. S3A,B). To test whether *β1-int* RNAi affected the number of mitotic cells during regeneration, we performed immunofluorescence analysis of head, trunk and tail fragments at eight different time points after amputation (Fig. 1B; Fig. S3C) using the anti-phospho-histone H3 (Ser10) (H3P) antibody, which specifically labels mitotic cells (Hendzel et al., 1997; Wei et al., 1999). The mitotic amputation response in wild-type *S. mediterranea* is characterized by a wound-induced global increase in mitotic cells around 6 h after amputation (hpa), a drop after 18 hpa, and a regeneration site-specific increase around 48 hpa (Wenemoser and Reddien, 2010). Strikingly, in *β1-int* RNAi fragments, mitotic counts were comparable to control (ctrl) animals during the first 2 days of regeneration but were significantly elevated during all later time points tested (Fig. 1B; Fig. S3C). In homeostatic planarians at 25 days post injection (dpi) with dsRNAs against *β1-int*, we did not detect any change in mitotic cell number. This was despite the onset of head regression and the formation of lesions, possibly caused by increased levels of apoptosis (Fig. S4A-D). Our data indicate that *β1-int* expression is required to restrict the number of mitotic neoblasts during later stages of regeneration, when a blastema is already evident.

We next analyzed the expression of marker genes for neoblasts and various cell lineages in tissue fragments at different time points after amputation using quantitative real-time PCR (qPCR): *smedwi-1* (all neoblasts) (Reddien et al., 2005b), *NB.32.1g* (epidermal lineage) (Eisenhoffer et al., 2008; van Wolfswinkel et al., 2014), *coe* (neuronal lineage) (Cowles et al., 2013), *myoD* (muscle lineage) (Cowles et al., 2013; Scimone et al., 2014a) and *gata4/5/6* (gut lineage) (Martín-Durán and Romero, 2011; Wagner et al., 2011). Consistent with the increase in the number of mitotic cells, the expression of all markers was significantly elevated in regenerating *β1-int* RNAi tail fragments (Fig. 1C). Moreover, all lineage markers tested were strongly induced in cell populations from regenerating *β1-int* RNAi tail fragments that had been sorted based on high DNA content and small cell size [FACS X1 fraction: mainly neoblasts with double DNA content (4N)]. In contrast, expression of lineage markers in cell fractions enriched for neoblasts with single DNA content (2N) and irradiation-sensitive postmitotic progeny (X2 fraction), and in large irradiation-insensitive postmitotic cells (Xin fraction) was not altered when cells from *β1-int* RNAi fragments were compared with those from controls (Fig. 1D,E). This suggests that in regenerating *β1-int* RNAi planarians the pool of proliferating cells contains a higher number of lineage-restricted cells than that in regenerating control animals.

Next, we analyzed cells from regenerating planarians at 48 hpa, 5 dpa and 10 dpa by FACS (Fig. S5A,B). We did not detect any increase in the amount of cells with double DNA content at any time point, possibly due the small proportion of mitotic cells within the X1 cell fraction and the low sensitivity of this approach for detecting mitotic changes. However, the analysis revealed a significant increase in the percentage of X2 cells at 5 and 10 dpa (3-4%) (Fig. S5B). As the X2 fraction consists mainly of post-mitotic neoblast progeny, these data support a model in which more lineage-restricted progenitor cells are made in *β1-int* RNAi planarians.

To confirm that neoblasts in these animals gave rise to higher numbers of lineage-restricted cells, we analyzed *smedwi-1^+^* neoblasts for the expression of the epidermal lineage marker *NB.32.1g*, which is rarely activated in these cells under normal conditions, but abundant in neoblast progeny of the epithelial lineage (Eisenhoffer et al., 2008; van Wolfswinkel et al., 2014). As neoblast numbers are difficult to quantify *in situ* due to their high abundance in intact planarians, we performed this analysis in *β1-int* RNAi planarians that had been subjected to sublethal γ-irradiation (12.5 Gy), which depletes most but not all neoblasts (Salvetti et al., 2009; Wagner et al., 2011). We analyzed *smedwi-1^+^* colonies formed from surviving neoblasts and their progeny and found that the proportion of *NB.32.1g^+^* cells of all *smedwi-1^+^* neoblasts in these colonies was indeed increased, from ∼1.5% in control animals to ∼3.5% in *β1-int* RNAi planarians (Fig. 1F,G). Together with the increased expression of marker genes of various lineage-restricted progenitor cell types and the increase in the number of X2 cells, these data suggest that more neoblasts undergo differentiation into lineage-restricted progenitor cells when planarians lack *β1-int* gene expression.

### Integrins are necessary for amputation-induced re-distribution of neoblasts

**Fig. 2.**
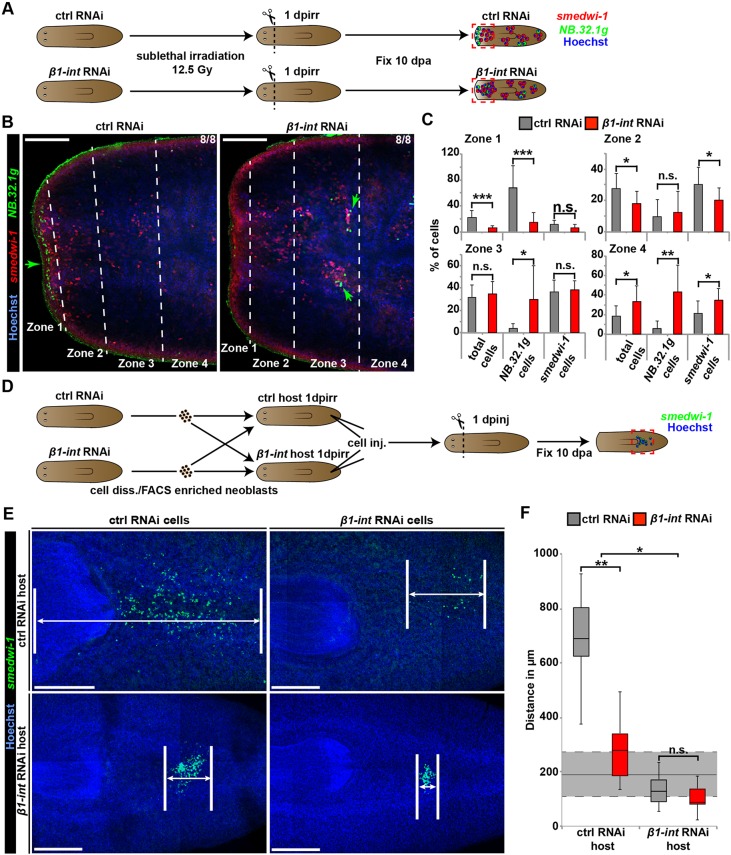
**Impaired cell migration after *β1-int* RNAi.** (A) Sublethal γ-irradiation assay with 12.5 Gy. See text for details. (B,C) Double FISH against *smedwi-1* (red) and *NB.32.1g* (green) reveals a significant net shift of neoblasts and *NB.32.1g^+^* progenitor cells (green arrows) towards the posterior in *β1-int* RNAi fragments 10 dpa. Fragments were segmented into four zones based on their distance from the anterior epidermis: Zone 1, up to 50 μm; Zone 2, 50-100 μm; Zone 3, 100-150 μm; Zone 4, >150 μm. The average percentage (+s.d.) *smedwi-1*^+^ and *NB.32.1g*^+^ cells out of the total cell number, of eight animals from each condition in every zone are plotted in C. (D) Cell transplantation assay. See text for details. (E,F) FISH against *smedwi-1* (green) reveals a significant decrease in neoblast dispersion for all *β1-int* RNAi combinations. Average distances between anterior- and posterior-most neoblast of seven animals are plotted in F in boxplots. The dark horizontal lines within the boxes represent the median for each condition, with the box representing the 25th and 75th percentiles and the whiskers indicating minimum and maximum values. The gray area marks the average distance (permanent line: ∼193.2 µm; *n*=7 animals) of transplanted ctrl neoblasts in uncut ctrl animals with s.d. (dashed lines: ∼±81.8 µm; *n*=7 animals). The composite image in E was generated using the customized tile scan function of Zeiss AxioVision software. Statistical significance (Student's *t*-test) is indicated (*P≤0.05; **P≤0.01; ***P≤0.001). DNA is blue (Hoechst). dpinj, days post injection; dpirr, days post irradiation; Gy, gray. Scale bars: 100 µm (B); 250 µm (E).

Neoblasts respond to tissue amputation by directed migration (Guedelhoefer and Sánchez Alvarado, 2012; Saló and Baguñà, 1985; Wolff and Dubois, 1948) and accumulate at the amputation site by 18 hpa (Wenemoser and Reddien, 2010). Moreover, cell migration is a process that is highly dependent on integrin-mediated adhesion (Friedl, 2004; Friedl et al., 1998). To investigate whether amputation-induced re-distribution of neoblasts could contribute to the blastema defect seen after *β1-int* RNAi, we analyzed the number of *NB.32.1g^+^* neoblasts in different regions of decapitated, sublethally irradiated ctrl and *β1-int* RNAi animals at 10 dpa (Salvetti et al., 2009; Wagner et al., 2011) using double fluorescence *in situ* hybridization (FISH) against *smedwi-1* and *NB.32.1g* (Fig. 2A). Consistent with previous reports on neoblast and progeny distribution in planarians (Eisenhoffer et al., 2008; Tu et al., 2015; van Wolfswinkel et al., 2014), most *NB.32.1g*^+^ cells were localized underneath the epidermis at the wound site of control animals (Zone 1), and a high density of *smedwi-1*^+^ cells was detected in a more posterior region (Zone 2). In contrast, in *β1-int* RNAi animals, the majority of *smedwi-1^+^* and *NB.32.1g^+^* cells were found further away from the wound site (Zones 3 and 4) (Fig. 2B,C), indicating a reduced ability of neoblasts to migrate towards the wound site.

To confirm this, we analyzed the ability of transplanted neoblasts to move towards an amputation wound. Therefore, we dissociated tissues from ctrl or *β1-int* RNAi planarians and live-sorted neoblast-enriched X1 cell populations by FACS (Wagner et al., 2011). After exposing ctrl or *β1-int* RNAi host animals to lethal γ-irradiation, we injected the FACS-sorted donor cells into the posterior parenchyma of the neoblast-depleted host planarians (Eisenhoffer et al., 2008; Hayashi et al., 2006; Reddien et al., 2005b) 1 day prior to head amputation. Interestingly, we discovered that the neoblast pool was drastically less spread at 10 dpa when ctrl RNAi cells were transplanted into *β1-int* RNAi instead of ctrl RNAi hosts (Fig. 2D-F). When *β1-int* RNAi cells were transplanted into ctrl RNAi hosts, this defect was milder but still significant. Although we cannot exclude the possibility that RNAi-mediated *β1-int* knockdown is transmitted from the host animal to the transplanted cells in the *gfp* RNAi donor/*β1-int* RNAi host set-up, these experiments clearly demonstrate that amputation-induced neoblast redistribution depends on the presence of integrins, and that this requirement is likely to be due to both autonomous and non-autonomous mechanisms. They further raise the possibility that the increase in lineage-restricted progenitor cells seen after *β1-int* RNAi (Fig. 1F) might be a consequence of their inability to migrate towards their target tissue where they would undergo terminal differentiation. A connection between neoblast migration and differentiation has been recently proposed (Abnave et al., 2016 preprint).

### Loss of *β1-integrin* function impairs the formation of organized tissues

Integrins mediate contacts between cells and ECM in many organisms by binding to ECM proteins such as fibronectin (FN), collagen (COL) and laminin (LN) (Campbell and Humphries, 2011; Humphries et al., 2006; Theocharis et al., 2016). To analyze the ability of *β1-int* RNAi cells to adhere to ECM proteins, we developed an *in vitro* adhesion assay (Fig. S6A). We labeled dissociated cells of ctrl and RNAi animals with calcein acetoxy-methyl-ester (Calcein-AM), a dye that stains live cells (Braut-Boucher et al., 1995; Fritzsche and Mandenius, 2010; Mariappan et al., 1999). After allowing cells to settle and attach to ECM-coated wells, we analyzed fluorescence intensity as a measure for the number of cells attached to the well surface after the removal of supernatant.

We found that, in wells coated with FN, COL or LN, fluorescence intensities from cells from *β1-int* RNAi planarians were lower (Fig. S6B) than those from control animals. In contrast, fluorescence intensities from *β1-int* RNAi cells in wells coated with poly-L-ornithine (p-ORN), a synthetic amino acid polymer mediating adhesion through electrostatic forces, were not changed. This suggests that cells are less capable of ECM protein binding when they lack integrins.

**Fig. 3.**
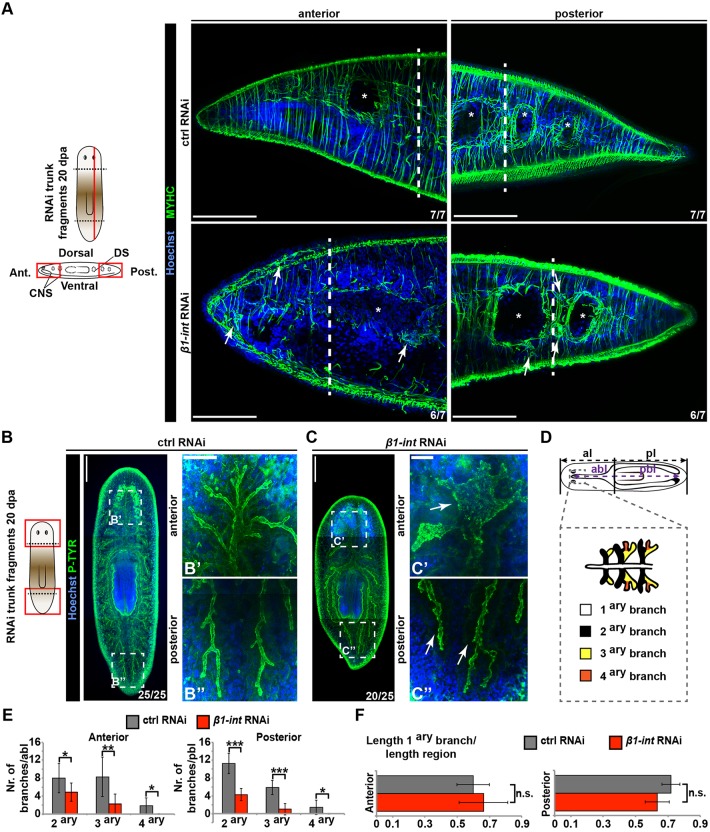
**Impaired muscle and gut regeneration in *β1-int* RNAi planarians.** (A) Anti-MYHC immunostaining (green) of sagittal sections of transversally amputated ctrl and *β1-int* RNAi trunk fragments at 20 dpa. Images show anterior and posterior regeneration sites. White arrows point to poorly structured muscle fibers; asterisks indicate gut lumen; white dashed line indicates approximate line of amputation. DS, digestive system; CNS, central nervous system; ant, anterior; post, posterior. (B-C″) Whole-mount immunostaining with an anti-PHOSPHOTYROSINE (P-TYR) antibody (green) indicate impaired branching of the gut (white arrows) in anterior and posterior blastemas of *β1*-*int* RNAi trunk fragments at 20 dpa compared with ctrl RNAi fragments. Composite images were generated using the customized tile scan function of Zeiss AxioVision software. High magnification images of the anterior and posterior regeneration blastema of *β1-int* or ctrl RNAi fragments (B′,B″,C′,C″) are taken from the boxed areas as indicated. (D) Scheme for evaluation of gut branches. abl, anterior branch length; al, anterior length; pbl, posterior branch length; pl, posterior length. (E) Average number of secondary (2ary), tertiary (3ary) and quaternary (4ary) per anterior or posterior primary branch length of eight fragments are plotted. (F) Length of primary (1ary) gut branches in relation to anterior or posterior animal length respectively is plotted. Error bars represent s.d. of eight fragments for each RNAi condition. Statistical significance (Student's *t*-test) is indicated (*P≤0.05; **P≤0.01; ***P≤0.001; n.s. not significant). DNA is blue (Hoechst). Scale bars: 100 µm (A); 250 µm (B,C); 50 µm (B′,B″,C′,C″).

Maintaining tissue organization is one important function of integrin-mediated cell-ECM adhesion (Boudreau and Jones, 1999). To get a first view on tissue organization in regeneration blastemas of *β1-int* RNAi planarians, we performed electron microscopy (EM) on high-pressure frozen transverse sections from anterior blastemas of tail fragments. Interestingly, we detected structural abnormalities in the blastema, where the subepidermal muscle layer appeared less packed than in control animals (Fig. S6B). To confirm this, we performed immunostaining with a myosin heavy chain (MYHC) antibody on regenerating *β1-int* RNAi planarians (transversal head and tail amputations and sagittal amputations) at 20 dpa (Cebrià et al., 1997). We found that the regenerating musculature was poorly organized and was characterized by the presence of many short-fibered muscle cells (Fig. 3A; Fig. S6D; Fig. S7A,B), whereas the musculature in pre-existing tissue seemed to be only mildly affected (Fig. S7A,B). This disorganization was also reflected in the more dispersed appearance of muscle cells expressing patterning factors (PCGs; Witchley et al., 2013) such as *slit* (Cebrià et al., 2007), *admp* (Gaviño and Reddien, 2011), *notum* (Petersen and Reddien, 2011), *sFRP-1* (Gurley et al., 2008; Petersen and Reddien, 2008) and *ndl-4* (Rink et al., 2009) (Fig. S8).

To test whether other prominent tissues were affected by the *β1-int* knockdown, we performed immunostaining against PHOSPHOTYROSINE (P-TYR), which labels the digestive system (Cebrià and Newmark, 2005), and RAPUNZEL (RPZ-1), a marker for goblet cells (Reuter et al., 2015), and quantified the number of gut branches as a measure of proper gut patterning (Barberán et al., 2016). Although lengths of the primary gut branches and proportions of goblet cells were not altered in anterior and posterior regions of regenerating *β1-int* RNAi planarians, the number of secondary, tertiary and quaternary branches was strongly reduced (Fig. 3B-F; Fig. S9A,B). Hence, in addition to altered neoblast behavior, the loss of integrins leads to a regeneration defect characterized by the disorganized formation of tissues, such as muscles and gut.

### *β1-integrin* RNAi causes formation of ectopic neural spheres

**Fig. 4.**
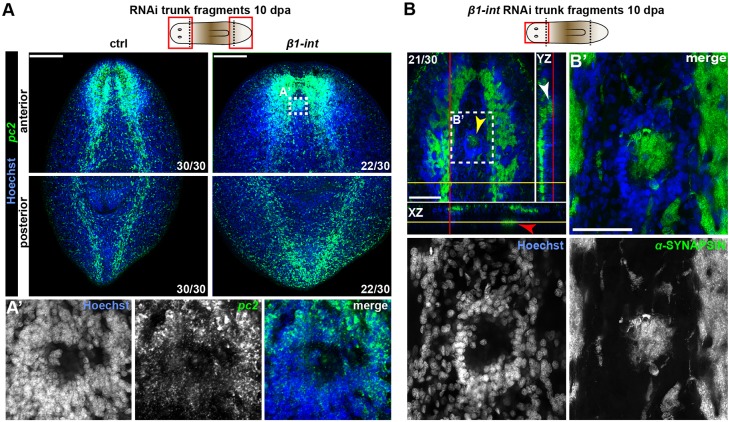
**Ectosphere formation in anterior blastemas of *β1-int* RNAi planarians.** (A,A′) FISH against *pc2* (green) on ctrl and *β1-int* RNAi trunk fragments at 10 dpa. White box indicates magnified area shown in A′, from another *z*-position within the ectosphere. The composite image in A′ was generated using the customized tile scan function of Zeiss AxioVision software. (B,B′) Anti**-**SYNAPSIN immunostaining (green) on *β1-int* RNAi trunk fragment at 10 dpa. Orthogonal view shows three ectospheres in the anterior blastema at different positions (yellow arrowhead in *xy* view, white arrowhead in *yz* view, red arrowhead in *xz* view). White box indicates magnified area shown in B′. DNA (Hoechst) is blue. Scale bars: 150 µm (A); 100 µm (B).

Using 5-ethynyl-2′-deoxyuridine (EdU) pulse-chasing (Zhu et al., 2015), we confirmed the ability of neoblasts to give rise to different lineages after *β1-int* RNAi as we detected EdU^+^ cells in the brain, in the gut and in the epidermis (Fig. S10A-H). Interestingly, however, we found higher numbers of newborn neurons and higher expression levels of the pan-neuronal marker gene *pc2* in *β1-int* RNAi animals (Fig. S10A,G,H), whereas markers for other tissues were unaffected or slightly reduced. Consistent with this, we detected accumulations of neurons in newly formed heads of *β1-int* RNAi fragments. These ectopic neural spheres (ectospheres) formed at a high frequency at random locations along the dorsal-ventral axis in 75% of all regenerating heads and were composed of *pc2^+^* neuronal cell bodies (Agata et al., 1998) on the outside and SYNAPSIN*^+^* tissue on the inside (Fig. 4A,B).

We found ectospheres only from 10 dpa, when the regenerating brain was already visible (Fig. S11A-E), and never detected them in posterior blastemas or in pre-existing tissues of laterally cut animals (Fig. S12A,B), suggesting that they form specifically in regenerating heads after brain regeneration has started. Interestingly, we also found ectospheres after *α-int-2* but not *α-int-4* RNAi, and double knockdown of *β1-int* with *α-int-2* led to an increased penetrance of this phenotype (Fig. S13A,B). These results support the notion that *α-int-2* is likely the major binding partner for *β1-int* during planarian regeneration.

### Ectosphere formation depends on neoblasts and anterior cues

**Fig. 5.**
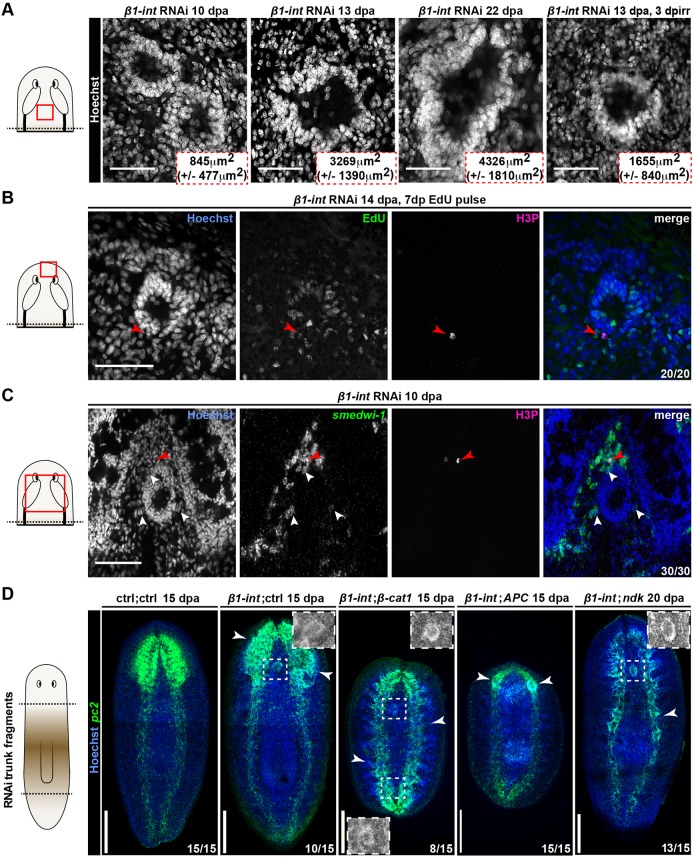
**Ectosphere formation depends on neoblasts and anterior cues.** (A) Ectospheres visualized by Hoechst staining (gray) in untreated or irradiated (3 dpirr) *β1-int* RNAi planarians. Average areas with s.d. of at least seven animals per time point are displayed. (B) EdU-pulse chase (green) of sphere-forming cells 7 days post injection (dpinj) and 14 dpa. The red arrowhead points to an anti-H3P^+^ mitotic cell. (C) FISH against *smedwi-1* (green) in combination with immunostaining against H3P (red arrowhead) reveals neoblasts in the periphery of ectospheres (white arrowheads) in anterior regenerating *β1-int* RNAi fragments 10 dpa. (D) Ectospheres, visualized by FISH against *pc2* (green) in regenerating trunk fragments of double RNAi planarians (ctrl;ctrl, *β1-int;ctrl*, *β1-int;β-cat1*, *β1-int;APC*, *β1-int;ndk*) at 15 or 20 dpa. White boxes highlight ectospheres in Hoechst (gray) channel. White arrowheads point to ectopic neural protrusions, described as brain primordium for *APC* RNAi animals (Iglesias et al., 2008). Red boxes in schemes illustrate areas of the images in the regeneration blastema of A-C. DNA (Hoechst) is blue. Composite images in D were generated using the customized tile scan function of Zeiss AxioVision software. Scale bars: 100 µm (A-C); 250 µm (D).

At 10 dpa, ectospheres had an average diameter of ∼49 µm (*n*=18; ±21 µm s.d.), which increased in size by a factor of 5 within 12 days. Analyzing ectospheres in *β1-int* RNAi animals after γ-irradiation (Fig. 5A) we observed decelerated growth of the ectospheres. We performed EdU pulse-chasing and immunofluorescence analysis for H3P to confirm that ectospheres were formed from newborn cells. We found most, if not all, ectosphere cells in *β1-int* RNAi planarians were EdU^+^. Although we detected mitotic cells in close proximity to ectospheres, we never found them within these structures (Fig. 5B,C). FISH against the stem cell marker *smedwi-1* confirmed the absence of neoblasts within ectospheres (Fig. 5C). Together with increased levels of neoblast and neuronal progeny markers after *β1-int* RNAi (Fig. 1C,E), these data indicate that ectospheres are composed of newborn neurons derived from neoblasts.

To test whether ectosphere formation requires anterior cues, we generated two-headed *β1-int* RNAi planarians by simultaneous knockdown of *β-catenin-1* (*β-cat1*) (Gurley et al., 2008; Iglesias et al., 2008; Petersen and Reddien, 2008). Strikingly, in *β-cat1;β1-int* double RNAi planarians, ectospheres formed also in ectopic head regions (Fig. 5D). Accordingly, the simultaneous knockdown of *APC*, a β-catenin antagonist knockdown of which results in two-tailed planarians (Gurley et al., 2008), prevented ectosphere formation (Fig. 5D). Simultaneous RNAi against *β1-int* and the putative fibroblast growth factor antagonist *nou-darake* (*ndk*), which restricts brain formation to the head region (Cebrià et al., 2002a), caused the induction of ectopic neural tissue along the anterior-posterior body axis, but not the formation of ectospheres outside the head region (Fig. 5D), demonstrating that ectopic neural tissue alone is not sufficient for ectosphere formation.

### Ectospheres are polar assemblies of various neural cells

**Fig. 6.**
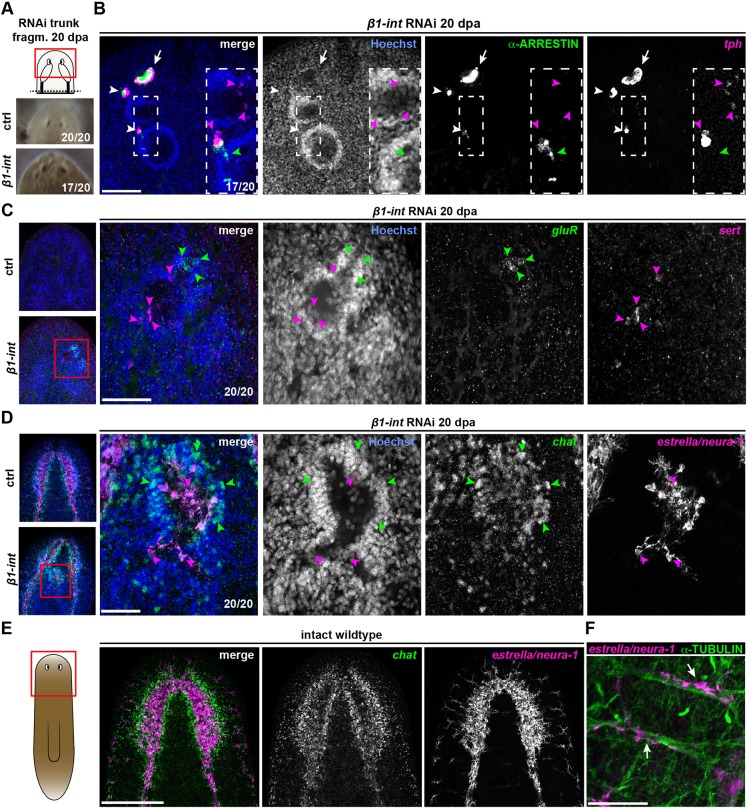
**Ectospheres are of multineural identity.** (A) Live images of anterior regeneration site of ctrl and *β1-int* RNAi trunk fragments at 20 dpa indicating ectopic eyes in *β1-int* RNAi animals. (B) Anti-ARRESTIN immunostaining (green; photoreceptor neurons) combined with FISH against *tph* (magenta) reveals the formation of ectopic eyespots (white arrowheads) with axonal projections towards ectospheres (green arrowheads), next to normal eyespots (white arrows) in *β1-int* RNAi fragments at 20 dpa. White boxes indicate magnified areas. Magenta arrowheads indicate *tph^+^* cells in ectospheres. (C,D) FISH on *β1-int* RNAi fragments at 20 dpa against neuronal markers *gluR*^+^ (green), *sert^+^* (magenta), *chat^+^* (green) and *estrella*/*neura-1^+^* (magenta). Magenta or green arrowheads indicate marker^+^ cells. (E) Double FISH against *estrella*/*neura-1* (magenta) and *chat* (green) on intact wild-type animals. (F) Anti-α-TUBULIN immunostaining (green) combined with FISH against *estrella*/*neura-1* (magenta). White arrows indicate cells in close proximity to α-TUBULIN^+^ axon bundles in wild-type animals. Red boxes in schemes illustrate areas of images of A-E. DNA is in blue (Hoechst). Scale bars: 100 µm (B); 50 µm (C,D,F); 250 µm (E).

**Fig. 7.**
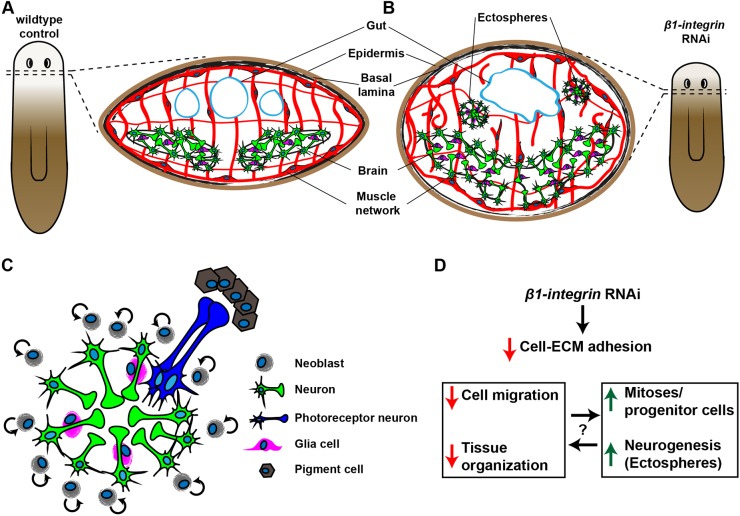
**The *β1-int* RNAi phenotype.** (A,B) Scheme of tissue organization in regenerating control (A) and *β1-int* RNAi fragments (B) at 20 dpa. *β1-int* RNAi planarians revealed severe regeneration defects of gut (blue line), brain (green and magenta) and musculature (red), and ectosphere formation. (C) Ectospheres are composed of neural cells including different neuronal subtypes (green) and glia cells (magenta). Occasionally they contact ectopic eyespots in their proximity [photoreceptor neurons (blue) and pigment cells (brown)]. Ectospheres are polar spheroids with neuronal cell bodies on the outside and axonal projections and glia cells on the inside, displaying a similar organization as the planarian brain. Proliferating neoblasts (gray) in the extracellular environment are likely to account for growth of ectospheres. (D) Summary of the effects of *β1-integrin* RNAi.

Fascinatingly, ectospheres made contacts with ectopic ARRESTIN*^+^* (Sakai et al., 2000) photoreceptor neurons that were assembled with pigment cells into an eye-like structure (Fig. 6A,B; Movie 1). Additionally, they contained regions of *tph^+^* (Nishimura et al., 2007) and *sert^+^* (März et al., 2013) serotonergic neurons, *gluR^+^* (Iglesias et al., 2008) glutamatergic neurons, and high numbers of *chat^+^* (Nishimura et al., 2010) cholinergic neurons (Fig. 6B,C; Movies 2,3). Furthermore, we found glia cells (Roberts-Galbraith et al., 2016; Wang et al., 2016), which are characterized by the expression of *Smed-neuron-associated-1* (*neura-1*)/*Smed-estrella* (*estrella*) (Roberts-Galbraith et al., 2016) and by their close association with axon bundles, inside the ectospheres (Fig. 6D-F). Hence, the composition of ectospheres suggests they could be primitive brain-like structures that depend on high levels of neurogenesis and anterior cues from the extracellular environment (Fig. 7C,D).

## DISCUSSION

Despite their prominent appearance in *β1-int* RNAi planarians and numerous publications reporting on proliferation, differentiation and patterning phenotypes in planarians (Almuedo-Castillo et al., 2014; Cebrià et al., 2002a; Fraguas et al., 2011; Gurley et al., 2008; Pearson and Sánchez Alvarado, 2010; Reddien et al., 2005a), ectospheres have not been described before. This suggests that their formation is specific to the loss of integrins or requires the concurrence of several defects (Fig. 7A-D). We found that ectospheres are dependent on cell proliferation and made of newborn neurons (Fig. 5A,B; Fig. 6). Together with the finding that regenerating *β1-int* RNAi planarians revealed more lineage-committed progenitor cells (Fig. 1), and particularly more neurons (Fig. S10), it is likely that excess neural progenitor cells are the major cell source for ectospheres. It is interesting to note that, although we detected more neurons in *β1-int* RNAi planarians than in control animals, excess progenitor cells for other lineages, such as the gut and the epidermis, did not result in excess formation of these tissues, suggesting that the conditions for survival and/or terminal differentiation in *β1-int* RNAi planarians are more favorable for the neural lineage.

Notably, in *β1-int* RNAi planarians ectospheres formed only in anterior regions (Fig. 5D) and they appeared to be polarized with *pc2*-expressing neuronal cell bodies on the outside and SYNAPSIN^+^ neuronal projections on the inside. This organization resembles the planarian brain, where regions of cell bodies and regions of axon bundles associated with glia cells are mutually exclusive (Fig. 4A,B; Fig. 6B-E). Strikingly, ectospheres made contacts with ectopic eyespot clusters composed of photoreceptor neurons and pigment cells (Fig. 6A,B; Movie 1), suggesting that ectospheres might be primitive mini-brains. However, the distribution of neurons positive for *gluR* mRNA, a marker commonly used to visualize the brain in planarians (Cebrià et al., 2002b), and other neuronal cell populations, appeared rather random (Fig. 6B-D) arguing against a brain-like regionalization in ectospheres.

Reduced neoblast migration and severe disorganization of regenerating tissues were further obvious phenotypes after *β1-int* RNAi. As cells from *β1-int* RNAi animals are less capable of binding to integrin-interacting ECM proteins (Fig. S6B), it is likely that the observed defects are based on reduced cell-ECM contacts. Inability of cells to bind ECM proteins would impair cell-ECM attachment and hence the formation of stable tissues. One of the tissues severely affected by *β1-int* RNAi is the regenerating body wall musculature, the cellular source for PCGs (Cebrià, 2016; Hill and Petersen, 2015; Owen et al., 2015; Reuter et al., 2015; Scimone et al., 2016; Witchley et al., 2013). The altered localization of some PCG-expressing muscle cells after loss of integrin function (Fig. S8) make it tempting to speculate that muscle disorganization resulting from reduced cell-ECM adhesion and/or apoptosis might cause aberrant signals that act non-autonomously on neoblasts to control their proliferation and differentiation behavior. This is supported by the relatively late increase in the number of mitotic cells and of lineage-restricted progenitor cells in regenerating *β1-int* RNAi planarians (Fig. 1B; Fig. S3C), suggesting that aberrant regeneration of a muscle-based positional coordinate system (Witchley et al., 2013) precedes these defects. As other organs, such as the gut, also poorly regenerated after *β1-int* RNAi, and the gut has been proposed to control neoblast proliferation non-autonomously (Forsthoefel et al., 2012), the gut and its underlying musculature are further candidates in contributing to the *β1-int* RNAi phenotype. However, one equally plausible explanation for the observed accumulation of lineage-restricted progenitor cells is their reduced migratory behavior (Fig. 2) after *β1-int* RNAi. Loss of integrin-mediated cell migration might inhibit these cells to reach their target tissue, leading to reduced terminal differentiation and the accumulation of cells in the progenitor state. Disruption of EGF receptor-1 signaling, for instance, which inhibits terminal differentiation into gut-resident cells, leads to increased numbers of *gata4/5/6^+^* gut progenitor cells (Barberán et al., 2016). Moreover, inhibition of epidermal differentiation after *egr-5* RNAi has been associated with increased *NB.32.1g^+^* progenitor cell production (Tu et al., 2015). These studies support both models, in which either signals from disorganized tissues instruct *β1-int* RNAi planarians on producing more progenitor cells, or a failure to migrate prevents terminal differentiation of progenitor cells and hence causes their accumulation in an undifferentiated state. In an alternative model, physical properties, such as tension and stiffness might influence neoblast behavior. Consistent with this, the body wall musculature of *β1-int* RNAi planarians appeared less compact (Fig. 3A; Fig. S6C,D; Fig. S7) and the morphology of these animals was altered towards a more round shape (Fig. S6D; Fig. S7B; Fig. S8A,D,E), suggesting that the body wall musculature is less capable of exerting force when *β1-int* expression is inhibited.

In summary, our study indicates that integrins are required for the organized formation of tissues during planarian regeneration and supports the notion that neoblasts rely on communication with an intact extracellular environment to control their behavior. Moreover, it suggests that excess neurogenesis can lead to *de novo* formation of ectopic mini-brain like structures given the availability of anterior cues and reduced integrin-mediated cell-ECM adhesion (Fig. 7C,D). Although an effect of poorly regenerated tissues, such as muscles and gut, on neoblast proliferation and differentiation is likely, our transplantation experiments demonstrate also a role of *β1-int* in facilitating amputation-induced neoblast migration (Fig. 2E). This migration defect might contribute to the manifestation of other observed regeneration defects, including ectosphere formation in anterior regions of regenerating planarians.

## MATERIALS AND METHODS

### Planarian culture and experiments

Planarians used in this study were from a clonal strain of the asexual *Schmidtea mediterranea* biotype BCN-10 kindly supplied by E. Saló (University of Barcelona, Spain), and maintained as described (Cebrià and Newmark, 2005). Animals were fed with calf liver and starved for at least 7 days prior to experiments.

### Irradiation of planarians

Planarians were lethally γ-irradiated with a total dose of 8000 rad (80 Gy) or sublethally irradiated with a dose of 1250 rad (12.5 Gy) in a Gammacell-40 Extractor (Nordion).

### RNAi

Injection of double-stranded RNA (dsRNA) was performed as described previously (Sandmann et al., 2011). In double-knockdown experiments, 1.5 μg/ml of each dsRNA (3 μg/ml total) was injected. Control animals (ctrl) were injected with dsRNA against *green fluorescent protein* (*gfp*). dsRNAs were synthesized according to Boutros et al. (2004). For regeneration experiments, planarians were either amputated pre- and post-pharyngeally or sagittally and observed at the time indicated. Live images were taken with a Leica M80 microscope. Primers for dsRNAs are listed in Table S2.

### Quantitative PCR

RNA extraction, cDNA synthesis and qPCR were carried out as described (Sandmann et al., 2011), and relative quantification of gene expression was calculated according to Pfaffl (2001). *gapdh* served as an internal reference gene. Primer sequences are listed in Table S2.

### *In situ* hybridization

*In situ* hybridization was carried out on whole-mount fixed planarians (WISH) and on sections as previously described (Nogi and Levin, 2005; Umesono et al., 1999) either manually or by using the InsituProVSi hybridization robot (Invatis). Images were taken with a Leica M165 FC microscope. Fluorescence *in situ* hybridization (FISH) was performed as described (Cebrià and Newmark, 2005; März et al., 2013) on whole animals and sections. For nuclear staining, Hoechst 33342 (Life Technologies) was used. For sections, the animals were passed through the *in situ* protocol until the first wash with Buffer 1 [100 mM maleic acid, 150 mM NaCl, 0.1% (v/v) Triton X-100]. Afterwards they were mounted in 3% agarose blocks and sectioned (70 μm) on a Leica VT 1200S vibratome before continuation of the protocol. FISH images were taken with a Zeiss laser-scanning microscope (LSM700) and processed and evaluated with Fiji (Schindelin et al., 2012). The LSM700 tile scan option, which automatically assembles tiled images to visualize larger structures, was applied for images in Fig. 2E; Fig. 3B,C; Fig. 4A; Fig. 5D; Fig. S4C,D; Fig. S6D; Fig. S10E; Fig. S11; Fig. S12 (sites of assembly are visible as thin lines). Primer sequences for *in situ* probe generation and references for marker genes are listed in Table S2.

### Immunohistochemistry

Immunostainings on whole animals or sections were carried out as previously described (Cebrià and Newmark, 2005). Antibodies used were: mouse anti-SYNAPSIN (3C11; 1:100; Developmental Studies Hybridoma Bank), rabbit anti-phospho-Histone H3 (H3P) (1:600; Millipore), mouse anti-ARRESTIN (VC-1; 1:15,000; kind gift from H. Orii, University of Hyogo, Japan), mouse anti-PHOSPHOTYROSINE (P-TYR-100; 1:500; Cell Signaling Technology) and mouse anti-TMUS-13 (MYHC; 1:500; kind gift from F. Cebrià; Cebrià et al., 1997). Secondary antibodies were Alexa Fluor 488 goat anti-mouse, Alexa Fluor 594 goat anti-mouse and Alexa Fluor 647 goat anti-rabbit (Thermo Fisher Scientific). Images were taken with a LSM700 (Zeiss) and processed with Fiji (Schindelin et al., 2012). H3P^+^ cells/mm^2^ were automatically counted using Fiji (Schindelin et al., 2012).

### Fluorescence-activated cell sorting

Planarian cell dissociation and gating was performed as described by Wagner et al. (2011) for live X1 enrichment. FACS was performed with the FACSAria Cell Sorter (BD Biosciences) and its respective software. X1 cell transplantations were performed as previously described (Böser et al., 2013). Planarians were fixed 7 days post X1 cell injection or cut pre-pharyngeally 1 day post X1 cell injection, prior to fixation at 10 dpa. Images were taken with a Zeiss laser-scanning microscope and processed with Fiji (Schindelin et al., 2012).

### EdU pulse chase

Approximately 100 nl of 0.05 mg/ml F-ara EdU [(2′*S*)-2′-deoxy-2′-fluoro-5-ethynyluridine; Sigma-Aldrich] was injected into the gut of RNAi animals 7 dpa for a 7-day chase (14 dpa). The animals were fixed according to the FISH protocol (März et al., 2013) and stained as previously described (Neef and Luedtke, 2011; Zhu et al., 2015). Images were taken with a LSM 700 (Zeiss) and processed with Fiji (Schindelin et al., 2012).

### TUNEL staining

TUNEL staining was performed as previously described (Newmark and Sánchez Alvarado, 2000; Pellettieri et al., 2010).

### *In vitro* cell adhesion assay

For the cell adhesion assay, flat-bottomed 96-well plates (Greiner) were coated with the ECM components collagen, fibronectin and laminin or poly-L-ornithine (all Sigma-Aldrich) at 4°C overnight: collagen: 10 mg/ml; fibronectin: 5 mg/ml; laminin: 2 mg/ml and p-L-ornithine: 5 mg/ml. Assay plates were washed twice with PBS and kept at room temperature. Biological triplicates of regenerating RNAi fragments at 20 dpa (ten fragments per replicate) were dissociated as described (Wagner et al., 2011). The cell concentration in the cell suspension was determined with a Neubauer cell chamber (Marienfeld Superior). A final concentration of 0.5 mg/ml Calcein-AM (Sigma) was added to the cell suspension (∼ 500,000 cells/ml) and carefully mixed. Afterwards 175 ml (∼87,000 cells) of cell suspension was pipetted into the coated wells and incubated at room temperature in the dark for 1 h. For the determination of background noise, some wells were incubated with Calcein-AM solution alone. After 1 h supernatants were carefully removed and wells were carefully washed with 1× CMFH solution [1× CMF (pH 7.3): 2.56 mM NaH_2_PO_4_·2H_2_O, 14.28 mM NaCl, 10.21 mM KCl, 9.42 mM NaHCO_3_; 1% bovine serum albumin; 0.5% glucose; 15 mM HEPES] and 1× PBS. Approximately 30 μl supernatant were left in each well and fluorescence intensity (from bottom) was measured at a wavelength of 517 nm with the BioTek Synergy-mix plate reader. The intensities were interpreted as a measure for the number of cells attached to the plate.

### Electron microscopy

Planarians were fixed by high pressure freezing followed by freeze substitution. For high pressure freezing, the samples were transferred in 6 mm aluminum planchettes, filled with 20% PVP in planarian water and were directly frozen with a high pressure freezer (HPM100, Leica). Samples were kept under liquid nitrogen until further processing. For freeze substitution, the samples were transferred in 1% OsO_4_, 0.2% uranylacetate, 5% water in acetone at −90 °C and stepwise dehydrated over 72 h (Helker et al., 2013). Samples embedded in Epon were sectioned transversally, 200 µm anterior to the eye region.

### Protein domain prediction and phylogenetic analysis

For predicting protein domains planarian integrin sequences and sequences from other organisms (best Blastp hit for respective planarian sequence), were used as an input for InterProsScan 5 (Jones et al., 2014). For the β-Integrin phylogenetic analysis we obtained amino acid sequences from previous publications and model organism-specific databases (Beckmann et al., 2012; Takada et al., 2007) (Table S1). Protein alignment was performed using MAFFT with E-INS-I strategy (Katoh et al., 2005; Katoh et al., 2002).

The maximum likelihoods were calculated using PhyML (Guindon et al., 2010) with the WAG model of amino acid evolution, four substitution rate categories, proportion of invariable sites and γ distribution parameter estimated from the dataset, and 100 bootstrap replicates. Trees were examined using FigTree (http://tree.bio.ed.ac.uk/software/figtree/) and rooted with integrin proteins.

### Accession numbers

Amino acid sequences of corresponding human genes were used for tblastn searches against transcriptome datasets (Brandl et al., 2015; Reuter et al., 2015). Best hits were confirmed using reciprocal BLAST. Accession numbers: *Smed-α-int-1* (KX024592), *Smed-α-int-2* (KU961519), *Smed-α-int-3* (KX024593), *Smed-α-int-4* (KU961520), *Smed-β1-int* (KU961518), *Smed-estrella/neura-1* (KX024594).
